# A qualitative examination of the impact of microgrants to promote physical activity among adolescents

**DOI:** 10.1186/1471-2458-14-1206

**Published:** 2014-11-22

**Authors:** Katherine A Tamminen, Guy Faulkner, Chad S G Witcher, John C Spence

**Affiliations:** Faculty of Kinesiology and Physical Education, University of Toronto, 55 Harbord St, Toronto, ON M5S 2W6 Canada; Faculty of Health Sciences, University of Lethbridge, 4401 University Drive, Lethbridge, Alberta T1K 3M4 Canada; Faculty of Physical Education and Recreation, University of Alberta, W1-34 Van Vliet Centre, Edmonton, AB T6G 2H9 Canada

**Keywords:** Microgrants, Case study, Adolescent, Physical activity, Sport, Qualitative

## Abstract

**Background:**

Microgrants are a mechanism for providing funding to community organizations or groups to support health initiatives. Little research to date has examined the use of microgrants in promoting physical activity (PA), and no studies have explored how microgrants may support PA promotion among adolescents. The purpose of this study was to explore the role of microgrants in enhancing PA opportunities for Canadian adolescents.

**Methods:**

Employing a case study approach, nine community organizations from across Canada were selected as cases providing sports and physical activities with the support of microgrant funding. Researchers visited each organization and conducted semi-structured interviews with 40 program participants (12–25 years of age, *M* = 16.3, *SD* = 2.6) and 17 adult organizers/instructors (23–57 years of age, *M =* 37.4, *SD =* 10.0). Interview transcripts were inductively and deductively coded to identify concepts and create a hierarchy of themes.

**Results:**

Analysis produced themes regarding participants’ perceptions of the *Funding, Running Programs and Events,* the *Impact of Program* (for the *Organization, Teen Participants,* and the *Community*)*.* Opportunities for PA programming would not have been possible without the microgrant funding. Microgrant funding was valuable in promoting PA for adolescents, and they afforded opportunities for adolescents to engage in new and/or nontraditional activities. In addition to promoting PA, the microgrants had benefits for participants and the community organizations including improved organizational capacity.

**Conclusions:**

Microgrants appear to be an effective mechanism for enhancing community capacity to provide PA opportunities for Canadian adolescents by helping to reduce financial barriers and empowering adolescents to take an active role in identifying and hosting new and creative PA events within their communities.

## Background

The majority of Canadian children and adolescents are insufficiently physically active to achieve health benefits [1]. Furthermore, a clear income gradient exists in which Canadian children from low-income households are less physically active than those from high-income households [2]. Among other factors such as body mass index, ethnicity, gender, self-efficacy, and social/parental barriers [3, 4], lack of opportunity or access to facilities [5, 6], and the costs associated with registration, equipment, and transportation are barriers to participation in organized physical activity (PA) and sport for children and adolescents [5, 7–9]. Thus, the reduction in social inequalities and disparities in access to PA have been identified as being a key principle of a population based approach to promoting PA [10].

Reducing the upfront costs of participation or access to services, and alleviating the burden of the associated expenses through tax deductions are two suggested approaches for addressing financial barriers to PA [11–14]. In the case of the latter, the evidence suggests that tax deduction programs and receiving a tax credit are inequitable by favoring those who can afford the expense and who pay taxes - as such they are ineffective economic instruments for promoting PA [15–18]. Conversely, subsidies for increasing PA participation among youth are thought to be more promising than tax deductions [19, 20]. However, the evidence is limited and would benefit from a more thorough examination of such programs.

Microgrants are a mechanism for providing funding through subsidies to community organizations or groups to encourage public participation in health initiatives [21]. They appear to be effective tools for enhancing community capacity, reducing inequalities, and testing new initiatives in the context of health promotion [21–26]. Among the few studies that have examined microgrants for programs that included some aspect of PA [22–24, 26], only one had a specific focus on supporting the promotion of PA [22]. Caperchione et al. described the Women’s Active Living Kits (WALK) Community Grants Scheme which distributed up to $1,500 (Australian Dollars) to 48 applicant groups across three states in Australia. The aim of the microgrants was increasing women’s participation in PA through the establishment of walking programs. Although their evaluation did not examine the effects of the microgrants at an organizational or individual level, the authors noted evidence of community partnerships being developed through the grant administration process.

Furthermore, there is no research examining microgrant funding for programs targeting children or adolescents. Thus, the introduction of a microgrants program in Canada to encourage adolescents ages 13 to 19 years to get physically active provides a unique opportunity to examine the potential role of such subsidies in promoting and facilitating PA. Specifically, ParticipACTION, a national not-for-profit Canadian organization dedicated to increasing PA in the Canadian population [27], supported by Coca-Cola Canada, established the Teen Challenge program (originally known as Sogo Active) which is a multi-year nationwide initiative that provides microgrants (Teen Physical Activity Grants) of up to $500 to registered community organizations to fund PA programs specifically for adolescents. Through a network of 13 provincial/territorial coordinators, the Teen Challenge has recruited and supported over 3950 community organizations across Canada since 2008 and distributed approximately 2520 grants, totalling over $2,000,000.00 in grants provided to community organizations.

Using the Teen Challenge program as an instrumental case study [28], the objective of this research was to explore the role of microgrants in enhancing PA opportunities for Canadian adolescents (http://www.participaction.com/teen-challenge/). Given limited research in this area, we adopted a qualitative approach in our investigation. This type of research comprises a wide range of approaches but it is usually characterized by rich description and narrative and is used to more closely represent the experience of participants. In particular, our goal was to develop a holistic and contextualized account of a purposefully sampled range of microgrant programs operating across Canada, and case study research is particularly well suited for such a goal [28]. Specifically, our research question was: what were the experiences of teen participants and program organizers who were recipients of microgrants? Through comparing and contrasting a collection of descriptive case studies we explored the impact of the grants on PA participation in the respective communities, and identified facilitators and barriers to their successful utilization.

## Method

### Case selection and participants

This evaluation is based on the first five years (2008–2013) of the Teen Challenge program which was coordinated by ParticipACTION; the study received ethical approval from the corresponding author’s Research Ethics Board. In consultation with the program administrators, multiple cases were purposefully selected [29] based on the following criteria: 1) reflect regional and geographic diversity among community organizations that had received Teen Physical Activity Grants, for example rural and urban areas within different provinces and territories; 2) represent a variety of organizations which had applied for funding, for example schools, community groups, traditional organized sports vs. PA programs; 3) represent a variety of adolescents, for example early/mid/late adolescents, urban/rural, male/female, adolescents with and without disabilities; 4) select organizers who had received funding twice or more; and, 5) identify organizers willing to participate in interviews that were able to recruit adolescent participants. A total of 13 cases were pre-selected based on these criteria and input and discussion from the team. To confirm interest in participating in the evaluation, community organizations were contacted by the program administrators to arrange an introductory phone call with a member of the research team, facilitated by ParticipACTION’s Projects Lead, in which the purpose of the study was explained and logistics clarified by explaining interview procedures and arranging site visit dates. Because four cases were unable to facilitate site visits, the final sample consisted of nine cases representing sports and physical activities such as breakdancing, community sport, school sport, skating, and skiing in British Columbia, Manitoba, Québec, Ontario, Newfoundland & Labrador, New Brunswick, Nova Scotia, Prince Edward Island, and the Yukon (see Table 1: Summary of cases). Forty adolescents and 17 adult organizers/instructors were interviewed. Youth participants ranged from 12-25 years of age^a^ (*M* = 16.3, *SD* = 2.6), and organizers ranged from 23-57 years of age (*M =* 37.4, *SD =* 10.0).

**Table 1:** **Summary of cases**

Province	Interviews	Organization type	Organization goals	Program demographics	Use of microgrant funding
Ontario	5 youth	Boxing program aimed to inspire and teach life skills about physical fitness, nutrition, community, social skills development, and mental skills.	Goal was to foster relationship building between participants and producing community champions.	Participants ranged from 6 to 18 years of age; majority of participants were between 12 to19 years of age. The participants were identified as “at risk” youth, youth from low socioeconomic status backgrounds, and new Canadians.	Funding was used for a four-week boxing/life skills program in which organizers taught youth life skills that are transferable into jobs and sports training. Provides an inclusive, safe environment, and teaches youth how to be their “true self.”
	3 organizers				
British Columbia	1 youth	Figure skating program.	Program was designed to support recreational and competitive figure skaters in a fun, friendly environment.	Participants ranged from 5 to 16 years of age; majority of participants were between 12 to16 years of age. Participants benefitted from funding due to low socioeconomic status.	Funding was used for skate-for-free day, bring a friend and get active day, skating scholarships for individuals demonstrating financial need, and strength training for skaters.
	1 organizer				
Yukon	5 youth	High school breakdancing program.	High school and program goals are to reduce bullying, improve peer acceptance, and promote active and healthy lifestyles.	Multicultural high school student population from grades 8–12 (13–17 years of age).	Breakdancing program developed through a partnership with the local dance studio; one of the instructors came to the high school to teach students about breakdancing. Program philosophy includes dancers in a dance circle which is supportive and where dancers can try out moves and learn from others.
	2 organizers				
Manitoba	9 youth	High school basketball program.	School/program objective was to try and promote PA and school pride while involving at-risk youth in after school activities to keep them safe and off the streets.	Inner-city high school serves the needs of students 13–18 of age from diverse cultural and economic backgrounds. Students are considered "at risk" or marginalized, low income, exposed to gangs and drugs, and includes new Canadians from all over the world.	Funding was used to purchase new basketball jerseys; in previous years students would re-use old jerseys or make their own.
	3 organizers				
Québec	5 youth	Youth community organization providing after-school and summer sports, cultural, and preventative programming.	Mission of the organization was to promote positive life skills development among all youth, but particularly for at-risk teens.	Participants ranged from 12–17 years of age; some participants also formed a ‘teen advisory board’ to provide input into which programs and events to organize for youth in the community.	Events included a ski and snowboard day to allow youth to try the activities for free; following the event the equipment was used to support the organization’s ski club, where teens could participate in skiing and snowboarding at the local ski hills. Additional funds were used to purchase multi-purpose sport equipment.
	2 organizers				
Nova Scotia	6 youth	High school breakdancing program.	School goals were to promote a diverse, progressive, educational community; to engage, challenge, and support students through the practice of respect and responsibility within a safe environment.	High school serving students 15–18 years of age.	Funding was used to support the school’s breakdancing club by hiring an instructor and support an annual competition to showcase students’ skills. The aim of the club was to develop confidence and physical skills in its members and to actively engage teens in an expressive art form.
	1 organizer				
Prince Edward Island	3 youth	Community fitness centre in partnership with local high school.	Program goal was to familiarize students with the fitness centre and help foster confidence with various modes of PA; to promote PA participation and to improve the health of participants.	High school in a small, rural farming community. Program participants were Grade 9 students (14 years of age).	Funding for students to participate in fitness sessions for free at the community fitness centre; funding covered drop-in costs as well as instruction/supervision by a personal trainer.
	2 organizers				
Newfoundland & Labrador	4 youth	Church group run by volunteers in small, rural, coastal community.	Providing recreational and social activities for youth within a faith-based structure.	Participants ranged from 11–18 years of age; participants from neighbouring communities were also invited to participate in activities.	Funding was used to purchase sport equipment and a ping pong table for youth at the church group; opportunities for recreation and PA were limited and the church group was the only provider of structured, organized activity for youth in the community.
	1 organizer				
Newfoundland & Labrador	2 youth	National, not-for-profit organization focusing on enhancing the quality of life of individuals with disabilities.	The goal of the family swim program was to provide a safe, supportive, comfortable environment to facilitate swimming skill development for individuals and their families.	Participants ranged from 11–25 years of age as well as family members (e.g., siblings, parents).	Funding covered the costs of booking a community pool to ensure participants did not incur costs related to participation. Free access removed potential financial barriers common among participants and their families. The family swimming program provided an important transitional link to other programs focused on developing competence among disabled individuals.
	2 organizers				

### Case site visit

Prior to visiting each case site, the interviewers spoke on the phone with the community organizer to gain insight about the program or event that was funded through the microgrant. Prior to arriving at the community organization, the researchers sent information letters and consent forms to the organizer to distribute to adolescents who might be interested in participating in an interview. Parent information letters and consent forms were provided to parents of adolescents. Upon arriving at the case sites, a member of the research team reviewed the study information with each participant prior to beginning the interview. Participants over the age of 18 provided informed consent prior to engaging in the interview and adolescents under the age of majority provided informed assent as well as signed letters of parental consent.

The interviewers spent one to three days in each location and learned about the community from the organizers and participants. In some cases, the interviewers had the opportunity to observe the adolescents participating in activities funded by the microgrants (e.g., watching breakdancing performances, watching sport practices), and in other cases they had the opportunity to view photographs and videos of the programs/events. This information was not collected for research purposes, but served to give the research team a better sense of the activities and the youth who had participated in the programs, and helped to familiarize them with the case, and contextualize the collected interview data.

Following each case site visit, the interviewers compiled a profile of each community organization (see Table 1 for a summary of cases) and noted their thoughts and impressions about the cases and the use of funding within each organization. The case summaries were sent to each community organizer for review and they were asked if the summary appropriately reflected their program or event and their organization. Organizers indicated that the summaries were reflective of their program and the use of their microgrant funds, and some organizers also provided additional details to support the case summary. For example, one case (Manitoba) was amended to reflect the demographic composition of the community and to state that their program serves the needs of marginalized and ‘at-risk’ adolescents.

### Interviews

Semi-structured interviews were conducted in a private room with each participant. In some cases a relative of the teen was present for the interview (e.g., some participants had developmental disabilities). The interviews included open-ended questions and were flexible to allow the participants to describe their experiences of the PA program or event. Main topics in the youth participant interviews included initial questions about PA (e.g., “tell me about the kinds of physical activities you do”), event/program awareness (e.g., “how did you find out about this event?” and “why did you participate in this program?”), teen experiences during the event/program (e.g., “what did you like/not like about the program?”), and wrap-up questions. Organizer and instructor interview topics included initial questions about PA within the community organization (e.g., “is there a mandate for your organization/program in terms of promoting PA?”), event/program implementation (e.g., “how did you become aware of the funding opportunity?” and “how did you recruit youth for this program?”), facilitators and barriers to running events/programs (e.g., “if you could design another program, what would you do differently?”, and capacity building/impact of the funding (e.g., “what impact did this program/funding have for your organization?”). Interviews with youth lasted 10–61 minutes (*M* = 28 min.) and interviews with adult organizers lasted 19–79 minutes (*M* = 46 min.). Adolescents received a $25 gift card for sporting goods or music and community organizations received a $100 gift card for their participation. Interviews were audio recorded and transcribed verbatim, yielding 819 pages of typed data (adults = 269 pages, adolescents = 550 pages). In the case of the French-language interviews in Québec, interviews were conducted and transcribed in French and translated into English. The researchers then checked the interviews with the audio recordings to ensure the meaning was accurately translated. Participants were assigned a code to maintain anonymity (e.g., YK P1 = Participant 1 from the Yukon; QC P8 = Participant 8 from Québec).

### Data analysis

Interview transcripts were imported into qualitative data analysis software (NVivo v.10) for organization and analysis. Analysis began by reading each interview in its entirety along with the case summaries to develop an understanding of each case, its participants, and the way in which the funding was used. Interviews were inductively coded to identify concepts and create an initial hierarchy of themes [29, 30]. The hierarchy of themes was used to deductively analyze subsequent interviews although any new inductively identified concepts were created and integrated into the hierarchy of themes. Thus, data analysis began inductively but became more deductive as analysis progressed. Community organizers’ and adolescents’ interviews from each site were coded together collectively as a case before moving onto the next case. For example, all the interviews from the Yukon case were analyzed as a group before analyzing the interviews from the Manitoba case to gain a better understanding of the case site and how the participants’ experiences were related. The results are organized into main themes and subthemes: *Funding (Overall value of funding; Flexible use of funds; Makes activity affordable), Running Programs and Events (Facilitators; Barriers or challenges), Impact of Program: Organization (Building credibility – leveraging funds; Building and strengthening partnerships; Sustainability and legacy), Impact of Program: Teen Participants (Increasing physical activity; Personal growth),* and *Impact of Program: Community (Community and family; Development of volunteers/teen leaders)* (see Table 2: Summary of themes). Since the focus of the study was on the implementation of the microgrant funding, the majority of the data presented below was reported by organizers, although some themes such as *Impact of Program: Teen Participants* and *Community* are supplemented with data from adolescents participating in the programs.

**Table 2:** **Summary of themes**

Theme	Subtheme	Example quote
Funding	Overall value of funding	*“Without the funding there's just kind of no program, right. There would still be Fridays at lunch and that would just be us, [but] there'd be no instruction. The kids would just be working on their own stuff and they'd have to get it from videos and when you don't have an instructor you just don't have a lot of momentum.”* (NS organizer P4)
	Flexible use of funds	*“It directly benefits the kids and you can use it for a lot of different things which is nice, too.”* (NS organizer P4)
	Makes activity affordable	*“That little seed money, it kind of helps us keep the youth engaged … it does help keep the kids coming back … The thing is for us to be able to keep the youth engaged that $500 really truly helps those little costs.”* (ON organizer P2)
Running Programs and Events	Facilitators	*“It wasn’t really until we got an instructor and then more people came …Having an instructor, I would say I improved a lot [compared to] the amount of effort I put in originally.”* (NS participant P6)
	Barriers or challenges	*“Our numbers were lower so … when we do it again that’s just what we have to think about is doing it more consistently.”* (YK organizer P5)
Impact of Program: Organization	Building credibility: leveraging funds	*“If help comes from another source, they [partners and granting agencies] will tend to give more.”* (QC organizer P6)
	Building and strengthening partnerships	*“Definitely it allowed us to strengthen them [partnerships with other sport organizations]. Minor hockey has way more money than we do. And they can offer so many more things than we can and normally we would not be able to participate in that kind of day with them because our costs are much higher.”* (BC organizer P1)
	Sustainability and legacy	*“It allowed us to continue the programs we already had by improving them to get more youth, more participants. We have basically expanded our network.”* (QC organizer P8)
Impact of Program: Teen Participants	Increasing physical activity	*“I didn’t really know a lot of different exercises to do with the weights like I do now after the program.”* (PEI participant P3)
	Personal growth (self-esteem, confidence, etc.)	*“[Breakdancing] is giving me opportunities and it has the potential to give me even greater opportunities, and it wouldn’t have happened without this program.”* (NS participant P5)
Impact of Program: Community	Community and family	*“We have had a couple of things where the parents will come on a try out free day … so we’ll say ‘OK yeah sure you want to come on with your kid that’s fine.’”* (BC organizer P2)
	Development of volunteers/teen leaders	*“…[Being involved in planning activities] we learn to be a little more independent, to know how to make decisions and to express ourselves … we represent a group, so we have to know how to express ourselves, to speak, to say what we think.”* (QC participant P4)

## Results

### Funding

#### Overall value of funding

Community organization leaders were overwhelmingly positive about the value of the Teen Physical Activity Grants and they acknowledged the importance the funding had within their organization. One director began by saying: “We would not be able to provide those opportunities without the funding, it’s pretty simple. It just wouldn’t happen” (BC organizer P1). The athletic director of an inner-city high school elaborated on the value of the microgrants:

“It was funny, ’cause you think it’s not like we got thousands upon thousands of dollars, but even just a small amount made it possible because we were struggling with fundraising and we were struggling to get the money to do certain things … Whereas I think without that funding, some of those things would have had to be axed.” (MB organizer P8)

For programs which had multiple objectives such as sport, PA, arts, and culture, the funding allowed community organizations to maintain all aspects of their programming:

“I am not just saying this because it is an interview – it really allowed us to get materials that we could not have had … It permitted us to do things we couldn’t have done if we hadn’t had the subsidy, or perhaps we could have done it, but we wouldn’t have had money to do other things too … So, it allowed us to continue other artistic and cultural activities but also to keep our sport component which is super important.” (QC organizer P8)

#### Flexible use of funds

Community organization representatives identified that one of the major benefits of the microgrants was the ability to use the money flexibly within their organization: “they give you a lot of flexibility in what you want to spend it on to promote your idea to get kids active” (MB organizer P1). The microgrants allowed organizers to develop programs which were responsive to their community’s needs:

“I know that the money is coming from top down but just the way that [ParticipACTION] have really allowed it to happen from a grass roots place, organically is really great … having faith that people have great ideas and know what their community needs.” (YK organizer P5)

Organizers also spoke about the value of the microgrants as opportunities to try out new programs or activities, or to refine the programs or events within the organization:

“It gives you that ability to tweak things. And so for us we realized realistically 10 weeks with holidays is too long of a challenge … So that’s something I would not have known until we actually delivered the program … I think that’s what’s cool about [the microgrants] because they will help fund the same [program] again, and they will help, in a sense tweak it.” (ON organizer P2)

Organizers in Québec said that in addition to being able to try out new activities, they were also happy that they could use the funding to maintain programs that had been previously developed: “there is a reality, do not reinvent the wheel. There were two skiing [programs]; even if they were similar, this is not serious. If something works, it isn’t necessary to always innovate. We must do what young people like” (QC organizer P6). The microgrants were unique funding opportunities because they allowed community organizations to sustain previously-established programs/events, or organizations could develop a completely new PA program/event based upon the teen participants’ interests. This process of having the opportunity to pilot programs helped organizers to determine which programs were going to be viable and sustainable within their organization and to develop ‘best practices’ for running programs in the future. Thus, the flexible use of grant funds was considered a major strength of the microgrants.

#### Microgrants make activity affordable

Many of the community organizations that received funding served as the main providers of PA opportunities for adolescents in the community. For example, the leader of a teen group said: “there’s nothing here for them so I just felt bad about it” (NL organizer P7). In some communities the local high schools served as a provider of PA opportunities for adolescents, particularly for adolescents from low socioeconomic households: “I think that money is kind of a part of it because I know that dance is really expensive, and there are a lot of sports that are really expensive so a lot of kids don’t have enough money to do those things” (YK organizer P5). As a result, a common theme throughout the interviews was that sport and PA was often “available but not accessible” to adolescents (QC organizer P8). The idea of activities being available but not accessible was particularly evident for families of adolescents with disabilities in small communities in Newfoundland & Labrador. Program organizers said that a swimming program served as one of the only sources of PA for the adolescents: “a lot of kids don’t feel comfortable going into the regular stream programming. But there’s nothing else…a lack of funding available for these families for recreation is big as well” (NL organizer P9).

Consequently, the microgrants enabled community organizations to make PA affordable and accessible within the community: “By giving these young people a wide range of activities, the resulting impact provides accessibility” (QC organizer P6). A teen from Manitoba said that without the funding to subsidize the school’s program, “it would have been more of a financial strain on my parents because they don’t really have the most optimal jobs and I don’t work either so I wouldn’t have been able to pay for it” (MB participant P14).

### Running programs and events

#### Facilitators

Program organizers identified two key facilitators for running their programs/events. First, all the program organizers discussed the ease of applying for the Teen Physical Activity Grants and said the application process helped them run the program or event:

“I’ve applied for a lot of grants for a lot of things that require you to write so much and they’re looking for something very specific … I really like the process for this one a lot better.” (NS organizer P4)

The grant application process was also useful for new organizations that did not have much experience applying for grants:

“In terms of, like ParticipACTION [Teen Challenge], the biggest takeaway I have was that because of the challenge, the way they worded challenge, it’s helped us on the organizational side get organized. ‘Cause we’re a new charity, we have all these ideas but we didn’t know how to make it most effective, and also we didn’t know how to measure. So we were just writing a program every Saturday with no real focus. The challenges have actually helped us focus and they’ve actually helped us in terms of making it a little bit better.” (ON organizer P2)

Another facilitator was having motivated, supportive leaders and instructors as the ‘champions’ for running programs/events: “I think that once you get an outside instructor locked down it becomes easy. A regular outside person that has good rapport with the kids” (NS organizer P4). It was also important to ensure the organizers and instructors had the support necessary to continue running the programs or planning events:

“This is going to sound kind of silly but what helps to run the programs is to help keep the people running the programs from burning out … just making sure that anybody who is running these things has that kind of underlying support so that they don’t run out of energy to keep these activities going for the kids.” (MB organizer P8)

#### Barriers or challenges

The community organizers reported a number of general challenges to running PA programs, including a lack of space or infrastructure to host events/programs, difficulties in recruiting volunteers (i.e., parents) to run events/programs, and insufficient government funding for PA programming. First and foremost, the organizers indicated that PA programming was expensive and they lacked money to run programs or events, which was one reason they were appreciative of the Teen Physical Activity Grants: “One of our biggest complaints is cost obviously. Yeah I think if I had to put my finger on the biggest challenge it’s trying to keep the costs affordable” (BC organizer P1). For example, hiring instructors to lead teen programs was often expensive: “I’d love to have more money to be able to pay the artists who are coming in to teach the kids what they’re worth as opposed to what the going cheapy rate is, so I always feel bad about that” (NS organizer P4). Another organizer in Manitoba said that cost was a challenge in trying to provide PA programming to large groups of teens: “it does get expensive especially when you’re working with large numbers of people so we have been getting, trying to be very creative with our funding and got used equipment and so on. I think the more we remove those barriers I think the easier it is for kids to participate” (MB organizer P1).

Some community organizations also reported difficulty in recruiting and retaining teens for programs/events: “One of the challenges is getting kids. You know like if you start something and you have 50 people sign up, after 3 or 4 months you only have 10 people left, and that’s what always happens with me” (NS organizer P4). Organizers identified that increasing teen recruitment was one of their main objectives for the future: “we have been trying to recruit more, that’s always been our priority really so we would just continue to do that” (NL organizer P3). Conversely, other community organizations reported that running programs or events with the Teen Physical Activity Grants increased enrollment in their programs (see below: Sustainability and legacy). Organizers who reported strong enrollment said that they used targeted approaches to advertising the program/event through word of mouth, media such as radio announcements, social media including Facebook and Twitter accounts updated by youth participating in the program, and through advertisements in local newsletters. Community organizers said that engaging youth in the initial development and advertising of the programs/events was also a key strategy to overcome the challenge of recruiting and retaining teens.

### Impact of microgrants

The microgrants were valuable in promoting PA opportunities for adolescents in communities across Canada. However, the microgrants had value beyond providing opportunities for PA: we also identified ways in which the microgrants had an impact for the organization, for the individuals participating in the programs, and for the participants’ families and communities.

### Impact of microgrants: organizations

#### Building and strengthening partnerships

The microgrants allowed community organizations to build and strengthen partnerships with other groups in the community. For example, a skating club in British Columbia partnered with a minor hockey organization to run a ‘try it free’ skating day. The organizer said that without the grant funding, the skating club would not have been able to participate with other organizations to run the event:

“Definitely it allowed us to strengthen [partnerships with other organizations]. Minor hockey has way more money than we do. And they can offer so many more things than we can and normally we would not be able to participate in that kind of day with them because our costs are much higher.” (BC organizer P1)

The microgrants were used to maintain existing partnerships. For example, a community organizer from a fitness centre in Prince Edward Island sought funding to allow youth in grade nine to participate in a weekly fitness class to familiarize them with the fitness centre and teach them about PA and working out at a gym, which addressed the strength-training curriculum in the students’ physical education classes. The microgrant funding was also used to work with existing community partners to maximize the impact it could have. For example, organizers purchasing ski equipment had existing relationships with the manager of a sporting goods store, and they were able to purchase ski equipment and maximize their relationship with the store:

“I can know a sponsor-partner but if I don’t have any money to get an advantage from him, then there’s no point … Previously, I was working at Sport Expert and the boss, I know him well … so he gave us some discounts. So, we had a bit more. For example, for five hundred dollars, you can usually buy a pair of skis if you are lucky, so if it isn’t too expensive and on special … Anyway, we got a lot more than what we could with regular prices so that helped a lot. The money helped our ski club a lot.” (QC organizer P8)

### Building credibility: leveraging funds

The microgrants helped community organizations to build credibility that they were competent and able to secure and use grant funding successfully. One organizer said:

“… in terms of the granting world how it works is when you go to apply for these grants they will only give you what you’ve managed. So these little grants over time are helping us show we manage our money responsibly, we’re able to deliver these things with the money. It helps build credibility with these other granters. And now this is our 3rd year and we just got approved for a $10,000 grant from a church, and it looks like we might get some other funding from [other granting agencies]. I would say it does help in terms of building credibility.” (ON organizer P2)

This organizer also said that the microgrant funding could be used as leverage to obtain additional funding from other organizations: “if we’re able to get $500 from ParticipACTION [Teen Challenge] and $500 from the police, now we have $1,000 budget, the program is going to grow” (ON organizer P2). Similarly, organizers in Nova Scotia said that by getting smaller grants such as the microgrants, it generated more grant funding opportunities for community organizations:

“I think success in grants definitely creates more buzz and success in grants. I think people who offer grant money want to see that it's be used. They want to see it's going to be used the way you said it was going to be used. They like to see a product of some kind …there's definitely a buzz about grants and getting out there as long as you know you can do it. Then I think that one grant turns into another, turns into another, turns into another.” (NS organizer P4)

Thus, another benefit of the ParticipACTION Teen Challenge Grant Funding was the ability for the community organizations to apply for further grants. If a Teen Challenge Grant was used to successfully implement a PA program or event, the organizers were appreciative of the option to apply for additional Teen Challenge Grants to support the program over time.

Community organizers in Québec also noted the value in obtaining funding from the ParticipACTION Teen Challenge program and the importance of associating themselves with organizations with the same values and priority on PA: “We attempted to associate ourselves, like with ParticipACTION, to promote sports, PA” (QC organizer P6). The director of a skating program said that when advertising the ‘try it free’ days and a skating scholarship program, “we actually put this article in the paper stating that it was in partnership with Coca-Cola and ParticipACTION” (BC organizer P1). Thus, by obtaining funding from the ParticipACTION Teen Challenge program, community organizations built credibility around their mission and their ability to use funding to support PA programs for adolescents within the community.

### Organization sustainability and legacy

In some cases the microgrants were used to sustain programs and created a lasting legacy beyond the duration of the program or event. One of the grants was used to hold a ski and snowboard event in Québec and the money was used in part to purchase ski equipment for the event; the equipment was then used by the community organization’s ski club. The ski club had been a tradition in the community for nearly 30 years, although it had suffered from low attendance and high costs. “We want to keep it here because it’s a tradition. So we work very hard to keep it running. So, I think without the equipment maybe it wouldn’t be, it wouldn’t have been possible to keep it … So this allowed the club to continue” (QC organizer P8).

One high school received funding to purchase new basketball jerseys, which improved athletes’ enjoyment while participating on the team and instilled a sense of school pride: “the uniforms offered us a way to have a legacy to continue using them to actually give the kids a set that they’d be proud to wear, represent our school in other buildings and you know I just thought there was great value in that” (MB organizer P1). One teen spoke about how wearing ‘real’ basketball jerseys helped to improve others’ perceptions of the inner-city school: “Well everyone around the school and other schools they basically say that our school’s really ghetto, so the [new] uniforms don’t make us look really ghetto” (MB organizer P3).

Several community organizations also said that running the programs or events with the microgrants increased teen enrollment in their programs. For example, one organizer said, “it increased our membership which increased our income, which then increased our staffing and our equipment, and just overall just everything got better for everyone” (BC organizer P1).

### Impact of microgrants: teen participants

#### Increasing physical activity

The programs or events offered with the microgrant funding allowed adolescents opportunities to develop existing physical abilities or to be exposed to new physical activities: “I never used to be able to swim on my own without anybody really close to me, but now I can swim on my own and not have to worry that I’m going to drown” (NL participant P7). The majority of adolescents said that by trying new activities, they also improved their physical fitness:

“I’ve been watching [breakdancing] forever and I’ve never gotten a chance to learn it, now I do. And it was a big bonus that I got in shape, but it wasn’t really a factor that pushed me, because I wasn’t like ‘man I really need to get in shape.’ I was just kind of like, I really like this, I want to do this, and I happened to get in shape because of it.” (NS participant P5)

One community organizer said that exposing youth to various activities helped to reinforce the message that “you don’t have to be an athlete to be active” (PEI organizer P4). Furthermore, adolescents said that the programs funded by the microgrants enabled them to discover a passion for an activity they had never experienced before: “It gave me something to do that I like enjoy doing, like I never had anything else that I liked so much before. Does that make sense? I finally found something that I think I would be doing for a really long time” (YK participant P1).

As a result of adolescents developing their physical abilities and trying new activities, the programs made it possible for adolescents to become more physically active and develop healthy lifestyles: “I’ve definitely become more physically active, and that’s a big difference in a person’s life … it’s made me healthier, it’s made me more aware, so I make better choices, and that’s definitely changed my life by doing things like that” (PEI participant P2). A teen in Ontario said that without the boxing program, “I would be way less active, I would come home, play [video] games, do nothing” (ON participant P6). In Québec, a teen said that before playing sports at the youth centre, “I was not very energetic at all. I hardly did anything, only walking with my friends and we would talk. That was it. I did not do any sports, nothing. But, now things are different. A couple of my friends practice sport a lot, and we are active. It’s fun like that” (QC participant P2). Another teen in Nova Scotia said that as a result of the fitness program offered through the high school, “I got a lot more active and just said I’ll stay away from the couch, I’ll stay away from the computer, all that stuff, just other lifestyle choices that I made” (NS participant P2). He continued and said:

“I’ve gotten noticeably thinner so … people are just asking if I had gone on a diet, if I had been exercising more. I say yeah, I just got more active … I kind of feel proud that I’m not the person I used to be and I’m proud that they are noticing that I’m making an effort to change. So it’s a kind of confidence boost there.”

#### Personal growth (e.g., self-esteem, confidence, leadership)

The adolescents’ development of their physical abilities also changed the way they viewed themselves and felt about themselves. For example, some adolescents said that being more physically active helped them to feel better about their weight and their abilities:

“With sports I definitely lacked a lot of confidence. I mean even last year when I was in the sports I didn’t want to try a lot of things, different moves and things, tried to play it really safe … [Now] I will try new things, more difficult things. Yeah, [I] feel more confident on the team.” (PEI participant P3)

Another teen said that through his participation in the program he learned “it’s OK to be different, like being fat and skinny, or tall and short, ‘cause it doesn’t really measure your talent. You can be tall but not good at dribbling, or short and good at rebounding” (MB participant P5). A girl participating in a boxing/life skills program said,

“If I don’t do this program, I don’t think I would ever knew how to protect myself, and if I got bullied or raped or anything I wouldn’t know how to protect myself … So boxing give me a lot, and my body changed … before I was a little big and when I came to boxing, my body changed, so [did] my personality, I became better person.” (ON participant P3)

Thus, the development of physical abilities helped the adolescents to develop a positive self-image as a result of participating in the community organizations’ programs or events.

Adolescents participating in the PA challenges also reported that they gained self-esteem, confidence, leadership, and social skills:

“I feel I have more confidence … Whereas before I had a massive inferiority complex. I used to be incredibly hard on myself, I would push myself to be better than other people just to feel like I could be more successful in ways that they were or successful in ways that they weren’t. But now I’ve stopped comparing myself to others and it was about a month after I started [the program] that so I think there is a correlation there.” (NS participant P2)

He continued, saying “I’m much more confident with myself now, I would say that breakdancing has been a big part of that.” The adolescents’ personal growth was corroborated by the adult organizers and instructors: “their self-esteem changes and they become very proud of what they’re doing and ‘look at me, look at what I can do’” (BC participant P1).

The adolescents’ positive growth and social skills also transferred into other areas of their lives. For example, Michael (pseudonym) was a teen with a disability who participated in a swimming program, and his aunt reported that prior to engaging in the swimming program he would not have felt comfortable even participating in an interview about his experiences: “he’s not a loner anymore … this is different too, for him to talk to a stranger and answer questions … And I said how do you feel about that? And he said ‘that’s OK Aunt Susan’” (NL participant P6). In Michael’s case, participating in a social activity such as a swimming program helped him to become more comfortable interacting with strangers in everyday life. Another teen who participated in a dance program and was able to go on a trip with his classmates said “Coming from a different country to Canada where things are a lot different, at first I didn’t really know how to engage in some of this stuff but once I started joining … I kind of gained the confidence that I know I need to live in this foreign country so I kind of developed a lot of confidence and self-esteem” (MB participant P12).

### Impact of microgrants: community

#### Community and family

The microgrants had a broader impact on the communities beyond the adolescents or the organizations that benefitted from the funding. A breakdance program served as an opportunity to develop stronger community relationships with new Canadians: “creating stronger partnerships with the international community right, like with the Filipino community … I think we have the same opportunity around dance to do that [develop partnerships] because that’s another thing that we’re very, very interested in” (YK organizer P5). Some of the PA programs or events offered opportunities for families to engage in affordable PA together. Organizers in Newfoundland & Labrador said:

“The ability that [the grants] allow us, like I say, to offer a free program is a big plus because a lot of our families are financially strapped so this gives them an opportunity to partake, and not only our participants partake but other kids that they’re financially strapped to keep active. ‘Cause keeping active is not cheap, to be involved in sports and other recreational programs are not cheap, so the fact that we can allow this not only for our participants, for their family, is a big thing.” (NL organizer P9)

Similarly, organizers running a teen boxing program hoped to have an impact on PA and healthy lifestyles for adolescents’ families: “It’s all about inclusion and even if you’re like, ‘you know what, I want to bring my big sister, I want to bring my mom,’ bring your mom, she can work out too, we don’t care, right, it’s all about creating that blueprint for life, it’s like a whole family working out together” (ON organizer P2). Adolescents also said that their increased PA had a positive impact on their family members: “it kind of passed over to my family … we've all been kind of getting more physically active. So it's kind of like a domino effect, like this starts and then the rest follows” (PEI participant P2).

#### Development of volunteers/teen leaders

There were several examples of community organizations and events/programs supported by the microgrants contributing to the development of volunteers and teen leaders, which in turn benefitted the organizations as well as the wider community. In some cases, adolescents were informal peer leaders when teaching one another skills, such as in the case of the breakdancing programs in some communities. In other cases, adolescents had more formal volunteer and leadership roles, such as helping with fundraising and event organization. For example, organizers in Québec engaged their teen advisory board to provide input for PA programming and to run the ski and snowboard day at the youth centre. A community organization in Ontario involved adolescents in the process of leading boxing classes and also in writing grant reports following the completion of events or programs:

“The whole goal is for kids in the program to later come back and become mentors. So for example one of the programs we ran with ParticipACTION is called ‘my turn to coach.’ The coach designs a workout plan, they assign tasks, they do a report, they learn to invoice, and then they get an honorarium of $20. So they’re actually getting real life skills as well as sometimes cash.” (ON organizer P2)

Teen volunteers reported that they learned a variety of skills through volunteering with the community organizations:

“You learn how to volunteer, you learn how to organize things, you learn how to say ‘OK this time I need this,’ and you learn how to schedule, you learn penmanship, people are like ‘I can’t read your writing,’ you’re like ‘oh I better fix that.’ You learn typing skills, e-mail skills, language skills, you get more opportunities ’cause you’re talking to parents and you have to talk respectfully, but you have to assert yourself as a person of authority, so you learn that kind of thing.” (BC participant P2)

Adolescents also expressed that they enjoyed becoming more involved in their community through volunteering:

“It really helped me realize how much I enjoy being active in school, active in the community and extracurricular activities … I started joining other programs that would help out with things because I enjoy being part of something, so it helped me realize that I enjoy being part of something big really and helping and meeting new people.” (NS participant P6)

## Discussion

The objective of this study was to explore the role of microgrants for enhancing PA opportunities for Canadian adolescents. Overall, there was considerable consistency among participants across the cases in describing the positive impact of the grants in their local context. In several cases, opportunities for PA programming would simply not have been possible without funding. How organizations ‘stretched’ the relatively modest grants to achieve their goals and the creativity with which many grants were put to use were both remarkable and innovative.

There are clear socioeconomic disparities in PA among children in Canada [2, 6]. The financial costs associated with organized sport and PA programs are a significant barrier to children and youth from lower socioeconomic backgrounds [7]. The most evident benefit of the microgrants was in making PA both affordable and accessible for many participants. The flexibility with which the grants could be used, and the relative ease in applying for the grants, was also noteworthy. This latter point should be considered in light of the concerns and frustrations commonly expressed by smaller organizations regarding the extensive proposal writing and grant reporting often needed in accessing funding [31].

The microgrants had impacts both at an organizational level with community partners and an individual level for adolescents themselves. Any increases in PA at an individual level are unlikely if organizational capacity to support PA opportunities is not enhanced [32, 33]. Accordingly, the grants should be seen as targeting an increase in organizational capacity and the case studies highlighted several ways in which this was happening. Smith et al. [34] described three organizational capacity indicators including leadership (e.g., the process of developing partnerships, collaborations, and linkages within the community); political will/policy making (the process of developing vision, mission, and political will of the target community to implement and sustain a health initiative); and, infrastructure (e.g., the skills, knowledge, and resources for health promotion). In terms of leadership, several examples were found of partnerships and linkages being developed as a result of the microgrants. Several cases also fostered the development of volunteers and teen leaders. As an example of political will, some organizations aligned themselves with the values and priorities of ParticipACTION and there were several instances of funds being used creatively to sustain impact, for example by purchasing equipment which can be re-used after the initial event. Most evident were benefits in terms of infrastructure and the provision of equipment, staffing, and other resources. The use of the microgrants in leveraging other funding sources was critical for many of the organizations. Most noteworthy was the finding in some cases that accessing the grant allowed organizations to demonstrate a successful track record of funding which in turn led to future funding success. Overall, the cases demonstrated examples of capacity development to promote PA to adolescents. This is consistent with other research on microgrants involving adult populations [22–24, 26] and should be seen as an important outcome of the Teen Physical Activity Grants.

One strength of this qualitative research was that it sampled a broad range of cases across Canada, and a qualitative approach was valuable in highlighting the range of benefits and positive impacts that the microgrant funding had for organizations, participants, and communities/families, beyond promoting PA for adolescents. Many of the adolescents reported that they were more active as a result of the event or program funded by the microgrants, however caution is required in determining the impact of the microgrants on actual PA levels of participating adolescents due to our qualitative research design. Building on this qualitative study, objective measurement of PA is likely needed to accurately assess such impact, although an appropriate research design to test such effects is likely challenging for two main reasons: first, to objectively assess the impact of microgrant-funded programs on adolescents’ PA, it would be necessary to track adolescents’ activity before and after the implementation of a PA program supported by a microgrant, which would be logistically challenging considering it is not known which adolescents might participate in any given program; second, the cost associated with such an approach would likely be substantial. What can be clearly supported is the role of the grants in exposing adolescents to a range of new activities and opportunities. Such experience might increase the chances of those adolescents finding an activity that best suits their interests and abilities which is more likely to lead to sustained engagement. A critique of traditional physical education curricula and school based sport programs is an unbalanced emphasis upon competition and team games which are taught using a limited range of didactic pedagogic approaches [35]. The majority of cases demonstrated the use (and attractiveness) of non-traditional activities such as noncontact boxing, breakdancing, and snowboarding. In other cases, activities were those that may be more feasible as youth enter adulthood – for example, swimming and fitness training. We speculate that the use of microgrants in response to local demand where youth and organizers identify PA possibilities within their communities can provide adolescents with alternative ideas about what PA is and what it can be. Additionally, enhanced self-esteem and the development of leadership and social skills were common benefits associated with participation in the activities. This is in line with research demonstrating that sport and PA can promote positive psychosocial development among youth when the context and circumstances are conducive in facilitating those benefits [36].

The importance of a ‘champion’ in the community that seeks out funding, motivates participation, and engages adolescents was seen as a key factor in the success of the cases. However the sustainability of programs does become less assured if there is reliance on one individual. Despite the value of the microgrants, community organizers still faced challenges in terms of infrastructure, funding and volunteers to help run programs. Importantly, retaining adolescent participation was a challenge at some sites. Most organizations were attempting to examine how best to engage youth earlier in the development of the programmes and in reaching out to other youth using modes of communication such as social media which may be more resonant with adolescents. Participants provided a range of recommendations for other organizations in seeking funding and running programs including: 1) offering programs on a regular and consistent basis, such as running programs in the same location on a weekly basis instead of monthly; 2) engaging adolescents in program development by creating a teen advisory committee to discuss how funding should be used and to volunteer in running programs and preparing reports after the progam/event; 3) providing introductory sport and PA programs for beginners; 4) using funding to partner with local stores and businesses to provide discounted equipment or supplies for running programs/events; and 5) using funding to purchase resources that can last beyond the event or program and be re-purposed for future programs or physical activities within the organization.

Given calls for further research examining the use of microgrants in the promotion of PA [22], the current study contributes to a limited literature by providing a detailed account of how grants are being used in ways that enhance capacity to provide PA opportunities for adolescents. A strength of a multiple case study approach is that wider, often unintended outcomes of an intervention can be ascertained and several examples were found of the grants having a broader impact on the communities beyond the adolescents or the organizations that benefitted from the funding (e.g., building partnerships between organizations, building sustainable programs, developing volunteers/teen leaders). We purposefully selected multiple cases that had applied and received several grants. Accordingly, it cannot be inferred that all grant receiving organizations across Canada were as successful as those described here. Rather, the intention is to facilitate ‘naturalistic generalization’ by the reader in adding their own parts to the story and forming their own generalizations [28] based on the descriptions of the case studies (see Table 1).

Future evaluations of the ParticipACTION Teen Challenge should examine the extent to which the programs that receive Teen Physical Activity Grants are sustained over the long term. Thus, questions relating to infrastructure, programming, and impact on the PA of adolescents could be addressed. Along these lines, it would also be useful to examine more thoroughly the context in which the Teen Physical Activity Grants are more or less effective, for example based on location [rural/urban], within isolated communities, the population being served, and the programming of new or unique activities. Finally, gaining insight on the economic impact and organizations’ leveraging of other funding by offering such microgrants would be a valuable contribution to the literature and practice.

## Conclusion

Based on this analysis of nine case studies, modestly scaled microgrants appear to be an effective mechanism for enhancing community capacity to provide opportunities for Canadian adolescents to engage in PA. Microgrants can help reduce financial barriers and empower adolescents to take an active role in identifying and hosting new and creative PA events within their communities. These types of funding programs may play an important role in promoting and enabling PA across populations, and the Teen Physical Activity Grants were found to have positive impacts for the organizations, participants, and for the broader community. Thus, microgrants merit further consideration as a mechanism for PA promotion at a population level.

### Endnote

^a^Three participants ages 21, 22, and 25 took part in a swimming program for individuals with disabilities. The program focused on adolescents and their families, but organizers did not prevent older individuals from participating. We chose to include their experiences to represent the diversity of cases supported by the microgrant funding.

## Authors’ information

KT is an assistant professor in the Faculty of Kinesiology and Physical Education at the University of Toronto. GF is a professor in the Faculty of Kinesiology and Physical Education at the University of Toronto and a Canadian Institutes of Health Research-Public Health Agency of Canada (CIHR-PHAC) Chair in Applied Public Health. CW is an assistant professor in the Faculty of Health Sciences at the University of Lethbridge. JS is a professor at in the Faculty of Physical Education and Recreation at the University of Alberta.
